# Real-world implementation of non-endoscopic triage testing for Barrett’s oesophagus during COVID-19

**DOI:** 10.1093/qjmed/hcad093

**Published:** 2023-05-23

**Authors:** R Landy, S Killcoyne, C Tang, S Juniat, M O’Donovan, N Goel, M Gehrung, R C Fitzgerald

**Affiliations:** Division of Cancer Epidemiology and Genetics, Department of Health and Human Services, National Cancer Institute, National Institutes of Health, Bethesda, MD, USA; Cyted Ltd, 22 Station Road, Cambridge CB1 2JD, UK; Cyted Ltd, 22 Station Road, Cambridge CB1 2JD, UK; Cyted Ltd, 22 Station Road, Cambridge CB1 2JD, UK; Cyted Ltd, 22 Station Road, Cambridge CB1 2JD, UK; Department of Histopathology, Cambridge University Hospitals NHS Foundation Trust, Cambridge, UK; Cyted Ltd, 22 Station Road, Cambridge CB1 2JD, UK; Cyted Ltd, 22 Station Road, Cambridge CB1 2JD, UK; Department of Oncology, Early Cancer Institute, University of Cambridge, Cambridge CB2 0XZ, UK

## Abstract

**Background:**

The Coronavirus pandemic (COVID-19) curtailed endoscopy services, adding to diagnostic backlogs. Building on trial evidence for a non-endoscopic oesophageal cell collection device coupled with biomarkers (Cytosponge), an implementation pilot was launched for patients on waiting lists for reflux and Barrett’s oesophagus surveillance.

**Aims:**

(i) To review reflux referral patterns and Barrett’s surveillance practices. (ii) To evaluate the range of Cytosponge findings and impact on endoscopy services.

**Design and methods:**

Cytosponge data from centralized laboratory processing (trefoil factor 3 (TFF3) for intestinal metaplasia (IM), haematoxylin & eosin for cellular atypia and p53 for dysplasia) over a 2-year period were included.

**Results:**

A total of 10 577 procedures were performed in 61 hospitals in England and Scotland, of which 92.5% (*N* = 9784/10 577) were sufficient for analysis. In the reflux cohort (*N* = 4074 with gastro-oesophageal junction sampling), 14.7% had one or more positive biomarkers (TFF3: 13.6% (*N* = 550/4056), p53: 0.5% (21/3974), atypia: 1.5% (*N* = 63/4071)), requiring endoscopy. Among samples from individuals undergoing Barrett’s surveillance (*N* = 5710 with sufficient gland groups), TFF3-positivity increased with segment length (odds ratio = 1.37 per cm (95% confidence interval: 1.33–1.41, *P* < 0.001)). Some surveillance referrals (21.5%, *N* = 1175/5471) had ≤1 cm segment length, of which 65.9% (707/1073) were TFF3 negative. Of all surveillance procedures, 8.3% had dysplastic biomarkers (4.0% (*N* = 225/5630) for p53 and 7.6% (*N* = 430/5694) for atypia), increasing to 11.8% (*N* = 420/3552) in TFF3+ cases with confirmed IM and 19.7% (*N* = 58/294) in ultra-long segments.

**Conclusions:**

Cytosponge-biomarker tests enabled targeting of endoscopy services to higher-risk individuals, whereas those with TFF3 negative ultra-short segments could be reconsidered regarding their Barrett’s oesophagus status and surveillance requirements. Long-term follow-up will be important in these cohorts.

## Introduction

Earlier cancer diagnosis improves outcomes and is a priority for policy makers, funders of research and healthcare improvement programmes.^1–4^ New technologies will play a key role in improving earlier diagnosis.^5^^,^^6^ However, even when evidence exists to support a new technology, there is a gulf between clinical trials and implementation^7^ due to challenges in developing the infrastructure required outside of research trials, reluctance of some healthcare professionals to engage in change, as well as the lag in redesigning clinical pathways with appropriate referral and reimbursement mechanisms. However, implementation provides a valuable opportunity for generating high-quality, real-world evidence to help accelerate the pace of change.

Barrett’s oesophagus is a pre-cancerous condition that presents an opportunity to transform outcomes from late diagnosis of oesophageal adenocarcinoma. An estimated 3–6% of individuals with reflux symptoms have Barrett’s oesophagus; however, it is estimated that <20% of patients with Barrett’s oesophagus are diagnosed, and, as a result, most cases of oesophageal adenocarcinoma are diagnosed *de novo*, without the opportunity to prevent progression from Barrett’s oesophagus to oesophageal adenocarcinoma.^8–10^ When dysplasia and early cancer within Barrett’s oesophagus are identified, they can be treated effectively as an outpatient procedure using endoscopic therapy. This is in stark contrast to the <20% 5-year survival and morbid treatment in the majority of patients who present with symptomatic, invasive cancer.^11^^,^^12^ To increase the proportion of patients diagnosed early, we need easily accessible, minimally invasive and cost-effective diagnostic tools.

The standard diagnostic test for Barrett’s oesophagus involves endoscopy; however, the high prevalence (∼20%^13^^,^^14^) of reflux symptoms means that population-wide screening is extremely resource intensive. Furthermore, endoscopic biopsy is prone to sampling bias since early cancerous changes can be subtle and the diagnosis of dysplasia is subjective,^15–20^ resulting in some cancers being missed at endoscopy. Additionally, since most upper gastrointestinal endoscopies do not reveal any pathology, there is a significant unnecessary burden on patients and the healthcare system.^21^ To help overcome these issues, we developed a non-endoscopic, pan-oesophageal cell sampling device, the Cytosponge, which has minimal operator dependence, and, when coupled with biomarkers, can be used to identify those individuals at highest risk for Barrett’s and associated dysplasia/early cancer, respectively.^22–24^ Three biomarkers are applied to the cell sample: (i) trefoil factor 3 (TFF3) to identify goblet cells, the hallmark of intestinal metaplasia (IM) in Barrett’s oesophagus^25^ and the proximal stomach^26^; (ii) p53, the most prevalent biomarker for malignant transformation of Barrett’s; and (iii) haematoxylin & eosin (H&E) for cellular atypia, an indicator for inflammation or premalignant dysplasia.^23^ Clinical trial data have shown that Cytosponge-TFF3 is a sensitive and specific test for Barrett’s oesophagus^23^ and can diagnose 10 times more cases of Barrett’s oesophagus than general practice usual care.^24^ For surveillance of individuals with known Barrett’s oesophagus, atypia and p53 can identify prevalent dysplasia and help distinguish patients at high and low future risk and inform their follow-up interval.^22^

Due to endoscopy backlogs arising from the COVID-19 pandemic, Cytosponge was prioritized for implementation within the National Health Service (NHS), but rather than testing individuals with a history of reflux who might not otherwise receive an endoscopy, the focus shifted to testing individuals in the secondary care system who were either: (i) awaiting a routine endoscopy for assessment of reflux symptoms, in the absence of any alarm symptoms; or (ii) awaiting a Barrett’s surveillance endoscopy procedure, provided they had not had dysplasia on the previous endoscopy or previous endoscopic therapy.

The aim of this study was to review the range of findings from 2 years of diagnostic Cytosponge samples. The centralized processing (provided by Cyted Ltd) also provides an opportunity to review the profiles of patients referred for endoscopy, both those with reflux symptoms and those undergoing surveillance, across routine NHS services. These data provide valuable insights into future implementation of non-endoscopic technology for early cancer diagnosis beyond the COVID-19 pandemic.

## Methods

The analysis included all Cytosponge samples sent to a UK-based diagnostics laboratory (Cyted Ltd), between 01 September 2020 and 31 August 2022. These included implementation pilots in NHS Scotland, NHS England, NHS Trusts who commissioned services directly to assist with the COVID recovery, and the Innovate UK funded DELTA Project for implementation (SRCTN91655550). All patients signed an NHS consent form for their Cytosponge procedure.

**Figure 1.**
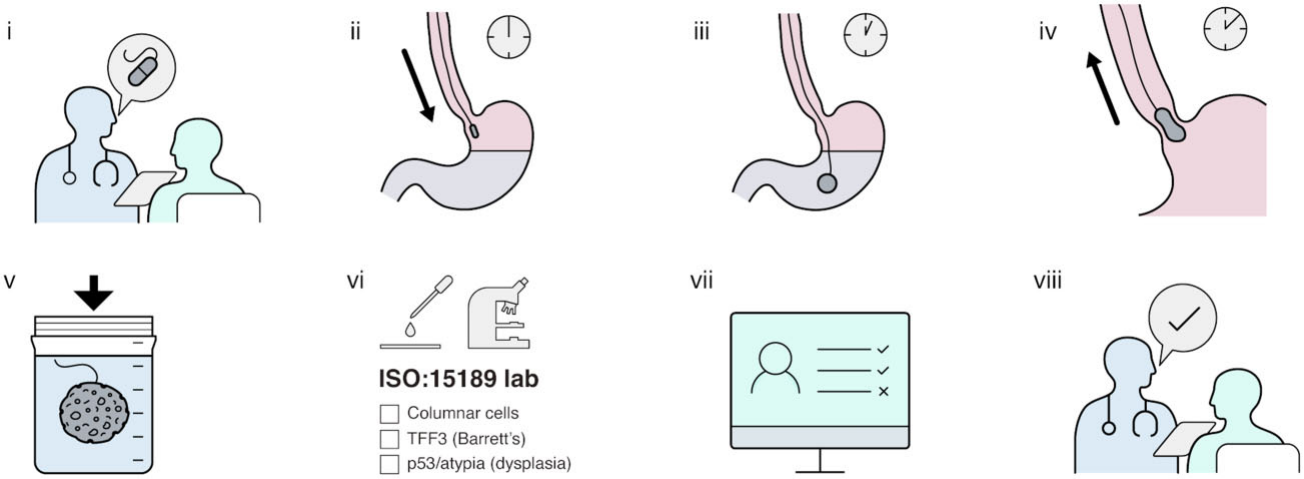
Cytosponge procedure and laboratory processing. (i) Clinician explains Cytosponge procedure to the patient. (ii) Cytosponge capsule tethered to thread is swallowed and enters the stomach. (iii) Capsule dissolves over 7 min and releases the spherical sponge. (iv) Clinician withdraws the sponge by pulling on the thread collecting cells along the oesophageal passage. (v) Retrieved sponge is placed into a preservative solution and transported to a central laboratory (Cyted Ltd). (vi) Cells are retrieved from the sponge, processed to FFPE sections, stained for columnar cells and atypia (H&E) and immunohistochemistry for TFF3 and p53 and processed to a digital image for review according to ISO 15189 regulations. (vii) A Cytopathologist reviews the images and generates a report. (viii) The result is communicated to the patient by the clinician.

Following administration Cytosponges were placed in BD SurePath Preservative Fluid, cells were retrieved and processed to paraffin blocks (formalin-fixed paraffin-embedded (FFPE)) as previously described.^24^ Atypia (including clear-cut dysplasia and atypia of unknown significance) was assessed on a superficial and deep section processed to an H&E slide. TFF3 immunohistochemistry (Roche 7F1.21 clone) and p53 immunohistochemistry (Roche Bp53-11 clone) were similarly performed on an adjacent superficial and deep subsequent FFPE slides. P53 staining with an intensity of 3 was considered significant, as previously published.^27^ Any sample with suspected atypia or aberrant p53 expression was reviewed by a second pathologist to confirm the findings, in accordance with best practice for reporting of dysplasia in biopsies^28^ (Figure 1).

For patients without a prior diagnosis of Barrett’s oesophagus, a sample was considered adequate if columnar cells were present in the sample, confirming that it had sampled as far as the proximal stomach. For individuals with known Barrett’s oesophagus, the pathologist also commented on whether gland groups were scant or sufficient for an assessment of dysplasia.

The pathology report included recommendations regarding whether an endoscopy was indicated and the degree of urgency. For all reports, clinicians were asked to treat the results in the context of the patient’s symptoms, especially since gastric pathology cannot be assessed from a Cytosponge sample.

Descriptive statistics were calculated to describe the population undergoing Cytosponge, by reason for the procedure. Differences in Cytosponge results (TFF3, p53 and atypia status) by sex and country (England vs Scotland) were assessed using chi-squared tests, and by age and length of Barrett’s segment using unadjusted logistic regression. When calculating the percentage testing positive for each biomarker, individuals with item missingness were excluded. When the results of multiple biomarker tests were combined (e.g. positive for p53 and/or atypia), any sample which was positive for at least one of these biomarkers was considered positive, and individuals with no positive results and some missing results were excluded from the denominator. Analyses were carried out in R v4.2.2.^29^

## Results

A total of 10 577 Cytosponge procedures were carried out in England and Scotland between 01 September 2020 and 31 August 2022. Of these, 7.2% (*N* = 764/10 577) of samples did not contain any columnar cells suggesting that the gastro-oesophageal junction (GOJ) or Barrett’s segment was not adequately sampled, and 0.3% (29/10 577) were not assessable due to food material—these individuals were recommended to undergo a repeat test or endoscopy. Hence, the 92.5% (*N* = 9784/10 577) of procedures which had a sufficient sample for analysis are the focus of this analysis for biomarker results, though demographic descriptions include all individuals undergoing Cytosponge. NHS England carried out 40.7% (*N* = 4305/10 577) of procedures, 43.9% (*N* = 4639/10 577) by NHS Scotland and the remaining 15.4% (*N* = 1633/10 577) by NHS Trusts who commissioned the service directly or as part of an implementation project called DELTA. Altogether 61 individual hospitals across England and Scotland administered Cytosponges (Supplementary Figure S1).

Of the 10 577 test procedures, 42.1% (*N* = 4456/10 577) occurred in individuals who were referred due to reflux symptoms in the absence of any red flags (weight loss, dysphagia, anaemia). These included 663 patients pro-actively screened due to a long-term prescription of heartburn medication as part of the DELTA project. The remaining 57.9% (*N* = 6121/10 577) of procedures were carried out for surveillance of known Barrett's (Table 1). Sixty-six per cent of Cytosponge-TFF3 tests had a turnaround time of ≤7 business days, with 99% reported within 14 business days, including double-reporting for patients with suspected dysplasia or early cancer.

**Table 1.** Demographic characteristics of individuals who had a Cytosponge sample collected as part of this study, by the reason for the Cytosponge, with biomarker results by length of pre-existing Barrett’s segment among individuals undergoing surveillance for known Barrett’s oesophagus

	Symptomatic		Surveillance		Total	
	*N*	%	*N*	%	*N*	%
Total	4456	100	6121	100	10 577	100
Sex						
Male	1932	43.4	4122	67.3	6054	57.2
Female	2369	53.2	1724	28.2	4093	38.7
Missing	155	3.5	275	4.5	430	4.1
Age group (years)						
<30	201	4.5	17	0.3	218	2.1
30–39	490	11.0	102	1.7	592	5.6
40–49	580	13.0	315	5.1	895	8.5
50–59	978	21.9	840	13.7	1818	17.2
60–69	894	20.1	1604	26.2	2498	23.6
70–79	602	13.5	1735	28.3	2337	22.1
80+	103	2.3	367	6.0	470	4.4
Missing	608	13.6	1141	18.6	1749	16.5
Country						
England	3264	73.2	2674	43.7	5938	56.1
Scotland	1192	26.8	3447	56.3	4639	43.9

	Surveillance											
Barrett’s length (Prague (M))	Overall		TFF3 positive^a^		P53 positive^a^		P53 positive restricted to TFF3 positive samples^a^		Atypia^a^		Atypia restricted to TFF3 positive samples^a^	
	*N*	%	*N*	%	*N*	%	*N*	%	*N*	%	*N*	%
≤1 cm	1175	19.2	366	34.1	13	1.2	10	2.8	41	3.8	25	6.8
2 cm	1136	18.6	555	54.6	26	2.6	23	4.1	55	5.3	39	7.0
3–5 cm	1876	30.6	1320	76.3	86	5.0	83	6.3	149	8.5	137	10.4
6–9 cm	931	15.2	765	87.7	55	6.3	54	7.1	104	11.8	98	12.9
10+ cm	353	5.8	296	87.6	21	6.3	21	7.1	57	16.6	56	19.0
Missing	650	10.6	266	44.8	8	2.4	7	2.6	24	4.0	19	7.1
Total	6121	100	3568	63.4	209	3.7	198	5.6	430	7.6	374	10.5

a Restricted to those with sufficient sample.

### Individuals with reflux symptoms

Over half of the individuals undergoing a Cytosponge procedure due to reflux symptoms were women (55.1%, *N* = 2369/4301), 18.0% (*N* = 691/3848) were aged <40 years and 18.3% (*N* = 705/3848) were ≥70 years (Table 1).

**Figure 2.**
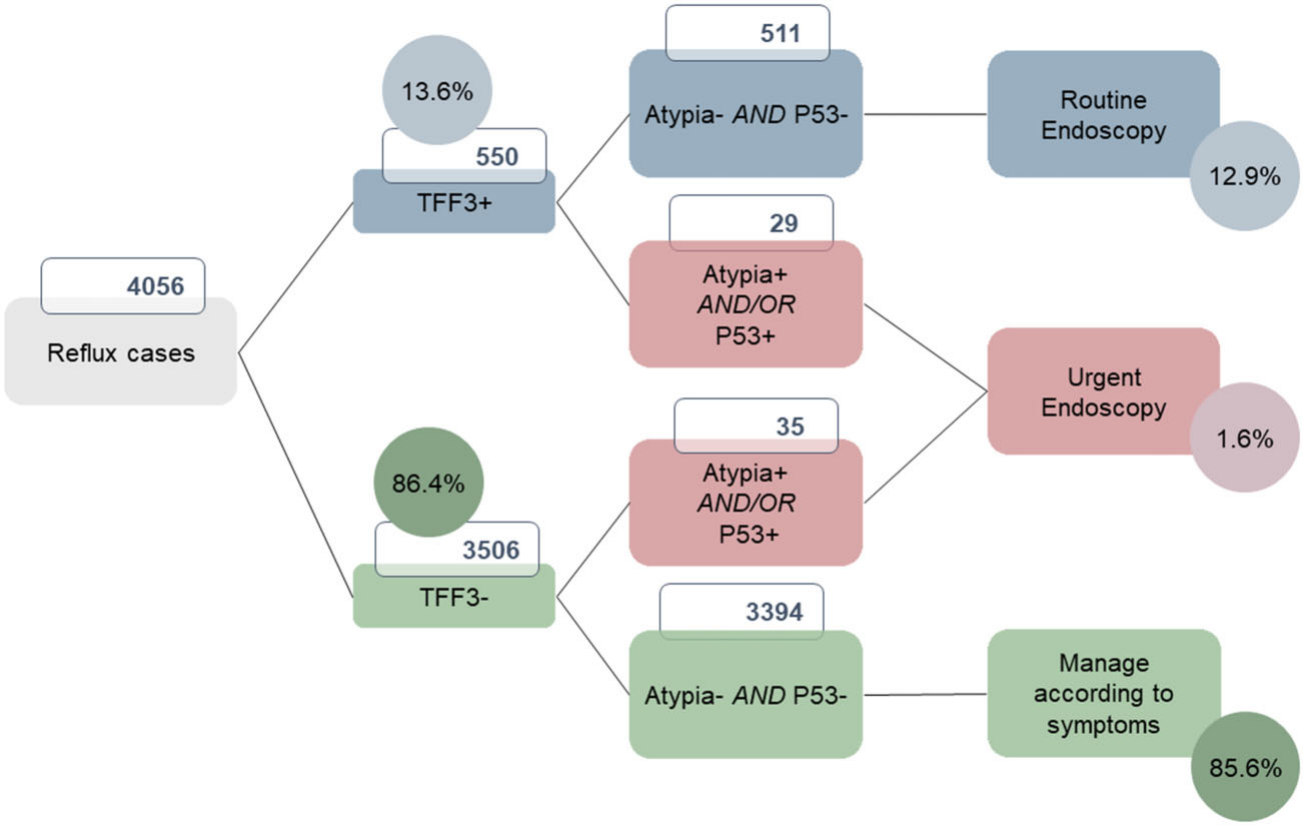
Flowchart showing biomarker results (TFF3, atypia and p53) for individuals with reflux, with clinical management recommendations. Note for 16 samples we could not retrieve the TFF3 result.

Around one-in-seven of the tests (13.6%) carried out due to reflux symptoms were TFF3-positive (*N* = 550/4056 with known TFF3 result), indicating the presence of IM which could originate from undiagnosed Barrett’s oesophagus, focal IM at the GOJ or IM of the cardia reflecting more extensive gastric IM (Figure 2, Supplementary Table S1). Of the TFF3-positive samples, 5.2% (*N* = 28/539, biomarker data missing for 11) had atypia and/or were p53-positive, suggesting that dysplasia or cancerous changes may be present (Supplementary Table S2).

The TFF3-positivity rate was higher in men than women (15.5% vs 11.9%, chi-squared = 10.2, *P* = 0.015), and increased with increasing age (odds ratio (OR) = 1.03 per year (95% confidence interval (CI): 1.03–1.04), *P* < 0.001), from 2.1% among individuals <30 years to 23.2% among individuals ≥80 years (Supplementary Table S1). The proportion with atypia (OR = 1.04 per year (95% CI: 1.02–1.06), *P* < 0.001) and p53-positivity (OR = 1.04 per year (95% CI: 1.01–1.08), *P* = 0.028) also increased with age. TFF3-positivity and atypia rates were significantly higher in Scotland compared to England (17.0% vs 12.3%, chi-squared = 14.2, *P* < 0.001, and 2.4% vs 1.2%, chi-squared = 6.3, *P* = 0.012, respectively). There was no significant difference in p53-positivity between Scotland and England (0.7% vs 0.5%, chi-squared = 0.2, *P* = 0.651). Some of the individuals (6.6%) (*N* = 269/4066) with reflux were found to have other abnormalities, including ulcer slough (*N* = 50).

### Individuals with known Barrett’s oesophagus undergoing surveillance

As expected, the individuals undergoing Barrett’s surveillance were predominantly men (70.5%, *N* = 4122/5846), with 70.4% (*N* = 3706/4980) aged ≥60 years, including 7.4% (*N* = 367/4980) ≥80 years. Among individuals with a reported Barrett’s segment length, 21.5% (*N* = 1175/5471) were ≤1 cm, 42.2% (*N* = 2311/5471) were <3 cm, 34.3% (*N* = 1876/5471) 3–5 cm and 23.5% (*N* = 1284/5471) >5 cm (Table 1).

**Figure 3.**
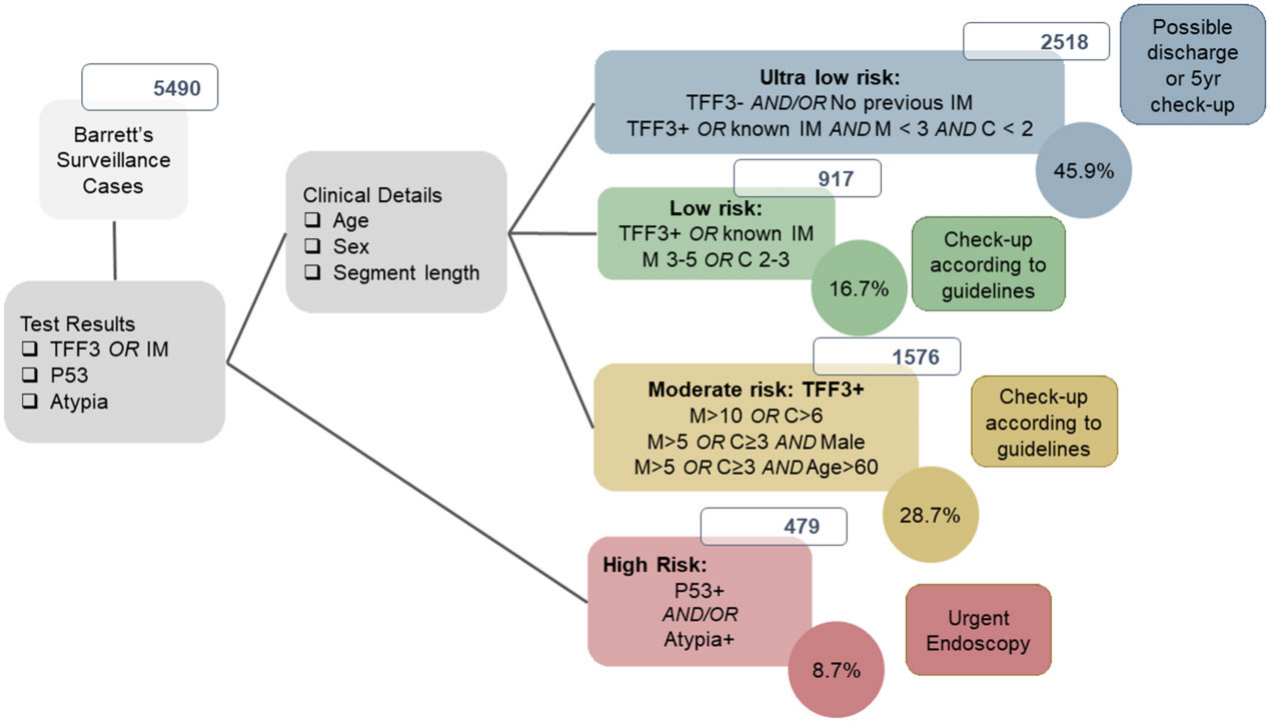
Risk categorization for samples collected from individuals undergoing surveillance for Barrett’s oesophagus. Note that 220 samples could not be allocated a risk category due to missing data on segment length.

In the UK guidelines (British Society of Gastroenterology), unlike all other International guidance, a definition of Barrett’s oesophagus does not require IM to be present.^28^ In all, 63.4% (*N* = 3568/5625) of samples in individuals undergoing surveillance for Barrett’s were TFF3-positive (Figure 3; Supplementary Table S3). TFF3-positivity increased with segment length (OR = 1.37 per cm (95% CI: 1.33–1.41, *P* < 0.001)), from 34.1% (*N* = 366/1073) among individuals with a reported Barrett’s segment length ≤1 cm, to 87.6% (*N* = 296/338) of individuals with a segment length ≥10 cm (Supplementary Table S3).

In total, 7.6% (*N* = 430/5694) of Cytosponge tests among individuals undergoing surveillance for Barrett’s had atypia suggesting inflammation or cancer, including 2.1% (*N* = 122/5694) with suspected high-grade dysplasia or early cancer (Supplementary Table S3). Similarly to TFF3, the proportion with atypia increased with increasing segment length (OR = 1.08 per cm, 95% CI: 1.05–1.10, *P* < 0.001), from 3.8% (*N* = 41/1086) for Barrett’s segment length ≤1 cm to 16.6% (*N* = 57/344) for those with segment length ≥10 cm, as did the proportion which were p53-positive (OR = 1.07 per cm (95% CI: 1.04–1.09, *P* < 0.001)), from 1.2% (*N* = 13/1073) of those with a segment length ≤1 cm to 6.3% (*N* = 21/335) of those with a segment length ≥10 cm (Supplementary Table S3). Eighty-one per cent (*N* = 162/201) of p53-positive tests in individuals with a known segment length were from individuals with a segment length ≥3 cm. When restricted to TFF3-positive samples, the rate of dysplastic biomarkers increased to 11.8% (*N* = 420/3552), increasing to 19.7% (*N* = 58/294) with TFF3-positive segments ≥10 cm (Table 1).

Additionally, 2.6% (*N* = 53/2051) of the TFF3-negative samples were p53-positive and/or had atypia in either columnar or squamous epithelial cells (Supplementary Table S4).

Using the previously published risk categorization^22^ which considers the patient’s age, sex and segment length as well as the p53 and atypia findings, 8.7% (*N* = 479/5490) of individuals were considered high risk and recommended to undergo endoscopy, with 28.7% (*N* = 1576/5490) considered moderate risk and the remaining 62.6% (*N* = 3435/5490) considered low or ultra-low risk, whereby the very short TFF3-negative cases may not fulfil the criteria for a Barrett’s diagnosis (Figure 3).

## Discussion

This article presents data collected from patients who underwent a Cytosponge test during 2020–2022, when NHS endoscopy services were limited due to COVID-19. Over 10 000 patients requiring surveillance of existing Barrett’s or triaging for reflux were tested over this period in real-world clinical practice. The centralized laboratory infrastructure was able to process and report samples within a 2-week period, with the majority returned within 7 days. This service can therefore benefit pathology, as well as endoscopy, bottlenecks.

For the patients with reflux symptoms referred to secondary care, the Cytosponge test identified 1.6%, with concerning high-risk findings requiring urgent endoscopy, and an additional 12.9% who were recommended routine endoscopy with a possible diagnosis of Barrett’s or gastric IM. Within this implementation pilot, clinicians were educated about how to interpret and utilize these results in a clinical care pathway. Patients with no findings on Cytosponge were checked, usually with a phone call, depending on the hospital service setup, to determine if their symptoms had resolved before deciding whether to refer on for endoscopy or discharge. Therefore, based on these results, we expect a substantial reduction in endoscopies required while maintaining high clinical safety. Longer-term follow-up will be required to evaluate the longer-term outcomes. It should be noted that although oesophageal adenocarcinoma and Barrett’s oesophagus are both more common in men than women,^30^ the majority of patients with reflux symptoms who received a Cytosponge were women, which likely reflects the increased health-seeking behaviours of women compared to men.^31^ Due to the higher rates in men, an organized pro-active screening programme would target men, and we note that the upcoming BEST4 trial^32^ of Cytosponge screening among individuals with gastro-oesophageal reflux disease and at least 6 months of prescriptions for acid-suppressant medications use in the past year will recruit men aged 55–79 years and women aged 65–79 years, due to the increased risk for men.

One of the striking findings is the breakdown of endoscopic and IM characteristics of patients undergoing Barrett’s surveillance across the UK. The British Society of Gastroenterology (BSG) guidelines for a diagnosis of Barrett’s oesophagus require a columnar lined segment >1 cm.^28^ Unlike European and US guidelines, there is no absolute requirement for IM to qualify for a diagnosis of Barrett’s if the segment is longer than 1 cm—this is predicated on the evidence for substantial sampling bias from endoscopic biopsy and a highly heterogeneous mucosa; however, for segments <3 cm with no IM, caution is advised in case this is a mistaken hiatus hernia. In this cohort, 21.5% were ≤1 cm and 42.2% were ≤3 cm; of these, the proportion who were TFF3-negative were 65.9% and 55.9%, respectively, suggesting they may have very focal IM that was not sampled or may not qualify for a diagnosis of Barrett’s oesophagus. In this implementation pilot programme, endoscopists were encouraged to review the TFF3-negative short cases for any previous evidence of IM, and if negative, to consider discharging. Hence, this centralized review of cases has provided an opportunity for cleansing of surveillance lists to avoid overdiagnosis and unnecessary monitoring.

Applying a risk-stratified decision tree for individuals undergoing surveillance for known Barrett’s oesophagus helps determine the priority for follow-up. In this cohort, none of whom had dysplasia on their last endoscopy prior to their Cytosponge, the proportion with IM (TFF3-positive) who tested positive for dysplastic biomarkers p53 and/or atypia was 11.8%. From published data, mainly from the USA, the per annum progression rate from non-dysplastic Barrett’s to low-grade dysplasia, high-grade dysplasia and cancer is 3.5–4%, 0.5–0.8% and 0.3–0.5%, respectively.^33–37^ Given that our previous prospective data suggest the positive predictive value of Cytosponge p53 and/or atypia was 31% for a diagnosis of dysplasia or cancer, this suggests that around 4% of TFF3-positive individuals undergoing surveillance have dysplasia or cancer, in line with what would be expected.^22^

In Europe, surveillance is intensified and performed in expert centres for patients with ultra-long segments >10 cm in view of their increased risk. Our data support this since the proportion of individuals with IM who have atypia and/or p53 over-expression was 19.7% (*N* = 58/294). The Cytosponge could provide a means for more intensive surveillance in this group.

There are some limitations. The Cytosponge is not always tolerated, and in around 8% of cases, the procedure had to be repeated due to a lack of sufficient sampling. Importantly, this analysis has focused on the laboratory findings, and the endoscopic correlations and long-term follow-up data are awaited. These will be critical to inform how non-endoscopic triage tools can be used in future.

Overall, this analysis offers evidence that non-endoscopic monitoring for patients can provide a promising method and real-world impact for triaging patients presenting with reflux symptoms and in patients undergoing Barrett’s surveillance. Future data will inform the frequency of testing, whether to alternate with endoscopy, and whether the diagnostic service could be based in the community setting to offer easier access for patients and to reduce healthcare costs. This implementation work has also shown how quickly one can pivot from research to a real-world setting—it is hoped that these valuable lessons in the setting of the COVID-19 pandemic will pave the way for more rapid adoption, pathway transformation and real-world testing of other emerging diagnostic technologies that promise to improve early cancer detection for patients.

## Supplementary Material

hcad093_Supplementary_Data

## Data Availability

Data will be made available upon reasonable request to the corresponding author.
