# Supplementary material for: Real-world implementation of non-endoscopic triage testing for Barrett’s oesophagus during COVID-19
Source: QJM. 2023 May 23;116(8):659–66. doi: 10.1093/qjmed/hcad093 (PMC10497181; doi:10.1093/qjmed/hcad093)
Supplement: hcad093_Supplementary_Data [file hcad093_supplementary_data.zip › hcad093_Supplementary_Data/Supplementary Tables_old.docx]

Supplement to **Real world implementation of non-endoscopic triage testing for Barrett’s oesophagus during COVID-19**

Rebecca Landy^1^, Sarah Killcoyne^2^, Charlene Tang^2^, Stephanie Juniat^2^, Maria O’Donovan^3^, Neha Goel^2^, Marcel Gehrung^2^, Rebecca C Fitzgerald^4^

**Supplementary Table 1**: Biomarker results for individuals who had an adequate Cytosponge due to reflux symptoms, by sex, age and country

|  | TFF3 positive | p53 positive | Atypia | TFF3, p53 and/or Atypia |
| --- | --- | --- | --- | --- |
|  | N (%) | N (%) | N (%) | N (%) |
| **Total** | 550/4056 (13.6) | 21/3974 (0.5) | 63/4071 (1.5) | 586/3980 (14.7) |
|  |  |  |  |  |
| **Sex** | | | | |
| Male | 269/1736 (15.5) | 13/1703 (0.8) | 33/1743 (1.9) | 287/1708 (16.8) |
| Female | 260/2178 (11.9) | 6/2131 (0.3) | 26/2186 (1.2) | 276/2132 (12.9) |
| Missing | 21/142 (14.8) | 2/140 (1.4) | 4/142 (2.8) | 23/140 (16.4) |
|  | | | | |
| **Age group (years)** | | | | |
| <30 | 4/188 (2.1) | 0/183 (0.0) | 1/188 (0.5) | 4/183 (2.2) |
| 30-39 | 31/464 (6.7) | 1/446 (0.2) | 5/465 (1.1) | 34/446 (7.6) |
| 40-49 | 55/538 (10.2) | 0/526 (0.0) | 4/539 (0.7) | 59/526 (11.2) |
| 50-59 | 121/898 (13.5) | 7/880 (0.8) | 13/901 (1.4) | 129/880 (14.7) |
| 60-69 | 142/809 (17.6) | 5/797 (0.6) | 18/816 (2.2) | 152/798 (19.0) |
| 70-79 | 108/525 (20.6) | 5/510 (1.0) | 13/525 (2.5) | 114/515 (22.1) |
| 80+ | 19/82 (23.2) | 0/79 (0.0) | 2/83 (2.4) | 20/80 (25.0) |
| Missing | 70/552 (12.7) | 3/553 (0.5) | 7/554 (1.3) | 74/552 (13.4) |
|  | | | | |
| **Country** | | | | |
| England | 367/2978 (12.3) | 14/2917 (0.5) | 37/2989 (1.2) | 387/2923 (13.2) |
| Scotland | 183/1078 (17.0) | 7/1057 (0.7) | 26/1082 (2.4) | 199/1057 (18.8) |

**Supplementary Table 2:** TFF3, atypia and p53 results among individuals with reflux symptoms

| TFF3 | Atypia | p53 | N | % |
| --- | --- | --- | --- | --- |
| Positive | Positive | Positive | 15 | 0.4 |
| Positive | Positive | Negative | 13 | 0.3 |
| Positive | Negative | Positive | 0 | 0.0 |
| Positive | Negative | Negative | 511 | 12.9 |
| Negative | Positive | Positive | 4 | 0.1 |
| Negative | Positive | Negative | 28 | 0.7 |
| Negative | Negative | Positive | 2 | 0.1 |
| Negative | Negative | Negative | 3394 | 85.6 |
| Total |  |  | 3967 |  |

**Supplementary Table 3**: TFF3, atypia and p53 results by Barrett’s segment length for individuals undergoing surveillance for known Barrett’s oesophagus

|  | [0-1 cm] | | (1-3 cm) | | [3-5 cm] | | [6-9 cm] | | [10+cm] | | missing length | | Total (including those with missing length) | |
| --- | --- | --- | --- | --- | --- | --- | --- | --- | --- | --- | --- | --- | --- | --- |
| Pathway | N | % | N | % | N | % | N | % | N | % | N | % | N | % |
| BO Surveillance (row %) | 1086 | 19% | 1033 | 18% | 1755 | 31% | 892 | 16% | 346 | 6% | 598 | 10% | 5710 | 100% |
|  |  |  |  |  |  |  |  |  |  |  |  |  |  |  |
| TFF3 |  |  |  |  |  |  |  |  |  |  |  |  |  |  |
| Negative | 707 | 66% | 462 | 45% | 411 | 24% | 107 | 12% | 42 | 12% | 328 | 55% | 2057 | 37% |
| Positive | 366 | 34% | 555 | 55% | 1320 | 76% | 765 | 88% | 296 | 88% | 266 | 45% | 3568 | 63.4% |
| Missing | 13 |  | 16 |  | 24 |  | 20 |  | 8 |  | 4 |  | 85 |  |
|  |  |  |  |  |  |  |  |  |  |  |  |  |  |  |
| Atypia |  |  |  |  |  |  |  |  |  |  |  |  |  |  |
| No | 1045 | 96% | 978 | 95% | 1601 | 91% | 781 | 88% | 287 | 83% | 572 | 96% | 5264 | 92% |
| Yes | 41 | 4% | 55 | 5% | 149 | 9% | 104 | 12% | 57 | 17% | 24 | 4% | 430 | 8% |
| Missing | 0 |  | 0 |  | 5 |  | 7 |  | 2 |  | 2 |  | 16 |  |
|  |  |  |  |  |  |  |  |  |  |  |  |  |  |  |
| p53 |  |  |  |  |  |  |  |  |  |  |  |  |  |  |
| Negative | 1060 | 99% | 993 | 97% | 1646 | 95% | 820 | 94% | 314 | 94% | 572 | 96% | 5405 | 96% |
| Positive | 13 | 1% | 26 | 3% | 86 | 5% | 55 | 6% | 21 | 6% | 24 | 4% | 225 | 4% |
| Missing | 13 |  | 14 |  | 23 |  | 17 |  | 11 |  | 2 |  | 80 |  |
|  |  |  |  |  |  |  |  |  |  |  |  |  |  |  |
| p53 and atypia positive | 10 | 1% | 17 | 2% | 65 | 4% | 43 | 5% | 19 | 6% | 6 | 1% | 160 | 3% |
| p53 positive and atypia negative | 3 | 0% | 9 | 1% | 18 | 1% | 11 | 1% | 1 | 0% | 2 | 0% | 44 | 1% |
| Atypia positive and p53 negative | 28 | 3% | 38 | 4% | 83 | 5% | 58 | 7% | 38 | 11% | 18 | 3% | 263 | 5% |
| Dysplasia suspected | 4 | 0% | 17 | 2% | 46 | 3% | 33 | 4% | 19 | 6% | 3 | 1% | 122 | 2% |

**Supplementary Table 4**: Atypia and p53 results for individuals who were TFF3-negative on Cytosponge

| Atypia | p53 | N | % |
| --- | --- | --- | --- |
| positive | positive | 160 | 2.8 |
| positive | negative | 263 | 4.7 |
| negative | positive | 44 | 0.8 |
| negative | negative | 5149 | 91.7 |
| Total |  | 5616 |  |

**Supplementary Figure 1**: The number of Cytosponge procedures carried out in each integrated care board in England and Scotland
