# Supplementary figures and images for: Real-world implementation of non-endoscopic triage testing for Barrett’s oesophagus during COVID-19
Source: QJM. 2023 May 23;116(8):659–66. doi: 10.1093/qjmed/hcad093 (PMC10497181; doi:10.1093/qjmed/hcad093)

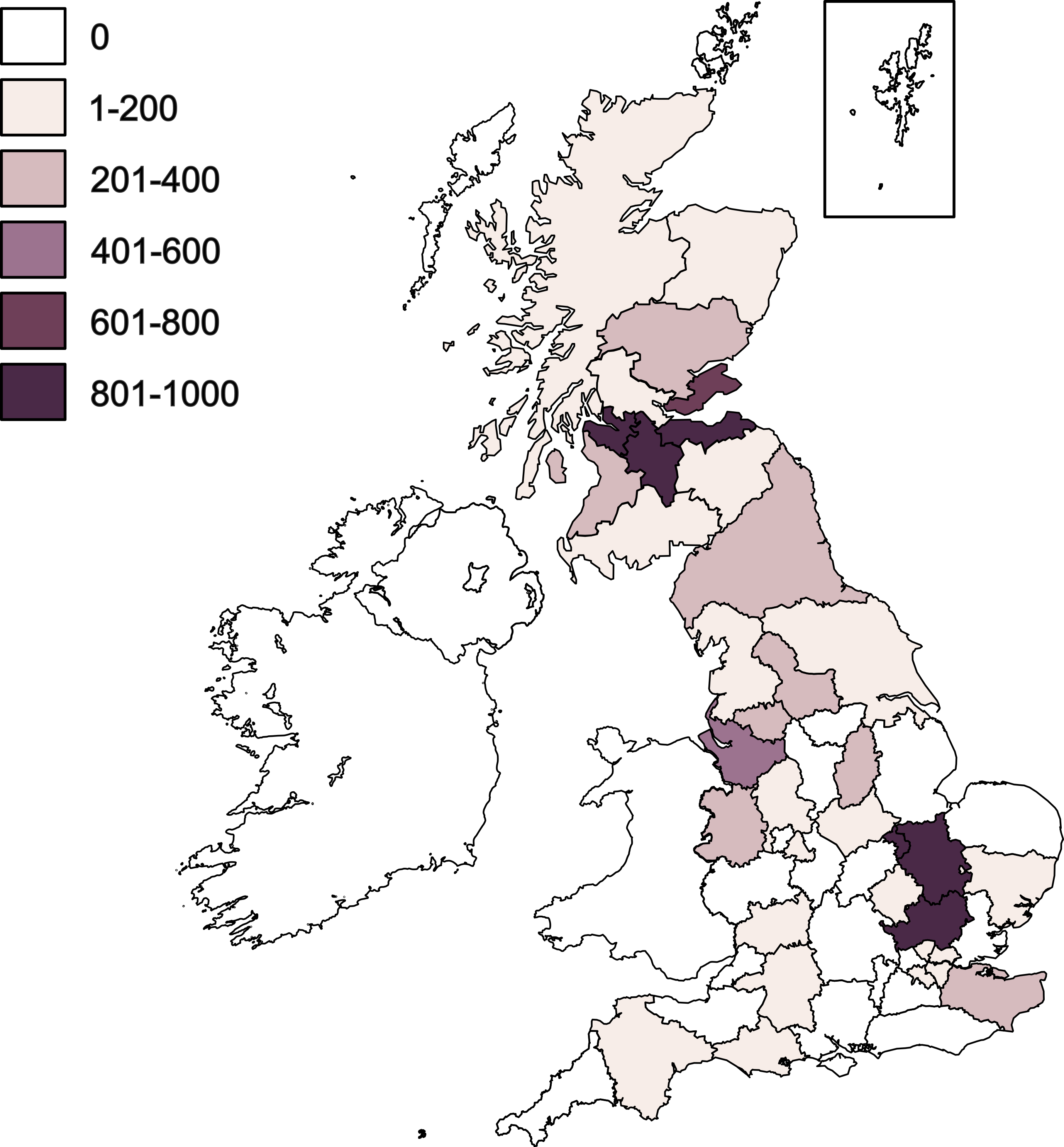

Supplement: hcad093_Supplementary_Data [file hcad093_supplementary_data.zip › hcad093_Supplementary_Data/Supplementary Figure 1.png]
